# Non-Coding RNAs as Prognostic Markers for Endometrial Cancer

**DOI:** 10.3390/ijms22063151

**Published:** 2021-03-19

**Authors:** Roberto Piergentili, Simona Zaami, Anna Franca Cavaliere, Fabrizio Signore, Giovanni Scambia, Alberto Mattei, Enrico Marinelli, Caterina Gulia, Federica Perelli

**Affiliations:** 1 Institute of Molecular Biology and Pathology, Italian National Research Council (CNR-IBPM), 00185 Rome, Italy; roberto.piergentili@cnr.it; 2 Department of Anatomical, Histological, Forensic and Orthopedic Sciences, “Sapienza” University of Rome, Viale Regina Elena 336, 00161 Rome, Italy; 3 Gynecology and Obstetric Department, Azienda USL Toscana Centro, Santo Stefano Hospital, 59100 Prato, Italy; annafranca.cavaliere@uslcentro.toscana.it; 4 Obstetrics and Gynecology Department, USL Roma2, Sant’Eugenio Hospital, 00144 Rome, Italy; fabrizio.signore@aslroma2.it; 5 Fondazione Policlinico Universitario A. Gemelli IRCCS, Gynecologic Oncology Unit, 00168 Rome, Italy; giovanni.scambia@policlinicogemelli.it; 6 Universita’ Cattolica Del Sacro Cuore, 00168 Rome, Italy; 7 Gynecology and Obstetric Department, Azienda USL Toscana Centro, Santa Maria Annunziata Hospital, 50012 Florence, Italy; alberto.mattei@uslcentro.toscana.it (A.M.); federica.perelli@uslcentro.toscana.it (F.P.); 8 Unit of Forensic Toxicology (UoFT), Department of Anatomical, Histological, Forensic and Orthopedic Sciences, Sapienza University, 00161 Rome, Italy; enrico.marinelli@uniroma1.it; 9 Department of Urology, Misericordia Hospital, 58100 Grosseto, Italy; 85cate@live.it

**Keywords:** endometrial cancer, molecular biology, non-coding RNA, biomarkers, prognostic factors

## Abstract

Endometrial cancer (EC) has been classified over the years, for prognostic and therapeutic purposes. In recent years, classification systems have been emerging not only based on EC clinical and pathological characteristics but also on its genetic and epigenetic features. Noncoding RNAs (ncRNAs) are emerging as promising markers in several cancer types, including EC, for which their prognostic value is currently under investigation and will likely integrate the present prognostic tools based on protein coding genes. This review aims to underline the importance of the genetic and epigenetic events in the EC tumorigenesis, by expounding upon the prognostic role of ncRNAs.

## 1. Introduction

Endometrial cancer (EC) is the most widespread gynecological tumor in developed countries. Its incidence is around seventy-nine cases per 100,000 women in Europe, with the average age at the time of diagnosis being sixty-two years [1,2].

Well-established risk factors have been identified: Lynch Syndrome and Cowden Syndrome genetic predisposition, polycystic ovary syndrome (PCOS), tamoxifen use, infertility, diabetes and obesity [3,4].

About one-third of patients have localized disease at the time of its first identification [1]. The prognosis for EC patients with early stage tumor (stages I and II) is mostly favorable. Currently, the treatment of patients with initial disease relies on risk factors reported within the European Society for Medical Oncology (ESMO), European Society of Gynaecological Oncology (ESGO), European SocieTy for Radiotherapy & Oncology (ESTRO) consensus published in 2016. Most of them can be submitted to surgery alone or followed by vaginal brachytherapy (BRT) or external beam radiation therapy (EBRT), adding platinum-based therapy in stage I high-risk and stage II patients [5].

The five-year overall survival (OS) is strictly stage-related, with a 95% OS for stage I women and 69% for stage II [1]. Nevertheless, some patients classified as low-risk have a relapse [6]. Patients affected by recurrent and advanced cancer (stage III or IV) have a poor prognosis, with a five-year OS related to metastatic disease between 15 to 17% [1]. This subset of patients is generally submitted to carboplatin and paclitaxel-based chemotherapy [5].

Prognostic factors for EC useful for management are represented by patient’s clinical–pathologic factors like age, stage, grading, and lymphovascular space invasion, and lymphovascular space invasion (LVSI).

Traditionally, EC has been divided in two pathogenetic groups, according to the Bokhman’s dualistic theory: endometrioid (Type 1) and non-endometrioid cancers (Type 2) [7]. Nearly 80% of EC type I patients had lower grade tumors, whereas 20% had high grade tumors [7]. The former are typically associated to a good prognosis, show high estrogen and progesterone receptors expression, and rarely get LVSI [7], while the latter are associated with poor prognosis, prevalence of high grade, low progesterone sensitivity, and high potential for LVSI [7].

Recently, The Cancer Genome Atlas (TCGA) described four EC groups based on genetic features: polymerase epsilon (POLE) mutated, hypermutated secondary to microsatellite instability (MSI), low copy number, and high copy number [8].

The current stratification of clinical–pathological risk implies that a significant percentage of patients are still too much or undertreated, with a large proportion of patients in the initial stage demonstrating distant metastases and some intermediate-risk patients who must undergo adjuvant therapies, to prevent a relapse, that will take only in a few cases [9].

For this reason, the trial “Post-Operative Radiation Therapy in Endometrial Carcinoma” (PORTEC)-4a is ongoing to define additional precise management strategies of early stage EC, by fostering the integration of molecular and clinical–pathological factors [10]. The authors defined three groups, according to their corresponding prognosis: (i) favorable, patients with POLE or microsatellite stable (MSS), p53 wild type (WT), and CTNNB1 WT; (ii) intermediate, patients with MSS, p53 WT, and CTNNB1 mutation; and (iii) unfavorable, patients with p53 mutation or >10% L1CAM expression [10].

Non-coding RNAs (ncRNAs) are constituted by transcripts of nucleotides with very little or no protein-coding capability. Their expression patterns in many malignant tumors consist of alterations which can promote or suppress tumorigenesis and cancer progression. They can regulate protein-coding organic phenomenon at epigenetic, transcriptional, post-transcriptional, and alternative levels [11].

Evidence shows that the anomalous expression of ncRNAs is associated to the prevalence, development, and prognosis of many cancers, and that they can be used as prognostic markers to guide the risk stratification of EC patients [12].

This review aims to summarize the genetic factors on which the current prognostic systems are based and to indicate the pathogenetic and the prognostic role of the ncRNAs, for the purpose of better defining tailored treatments and oncological surveillance on each EC patient.

## 2. The Genetics of EC

Recently, research has been focusing on the genetic characterization of human neoplasms, to better define prognosis and tailored therapies. EC represents one of the main tumors on which it has surfaced that genetic mutations and epigenetic modifications are the foundation of tumorigenesis. This is on account of its high incidence within the population and the discovery of prognostic factors that enable sound clinical management.

### 2.1. TCGA Classification

In 2013, the TCGA Research Network published, in *Nature*, the article titled “Integrated genomic characterization of endometrial carcinoma”. The authors performed a genomic and proteomic analysis of 373 EC and provided a diagnostic classification supporting the tumor’s molecular biology [8].

They defined four subtypes of EC according to genomic characteristics. Their molecular analysis showed that about 25% of G3 endometrioid EC have a molecular constitution like serous EC. The similarities between endometrioid and serous tumors led the authors to stated that genomic-based classification could result in improving management of EC patients, assuming the tendency to treat tumors with copy-number alterations with chemotherapy rather than radiation alone [8].

The authors reported four prognostic categories, as follows:

(1) POLE ultramutated

This group has the most favorable prognosis and a longer progression-free survival.

It is associated with endometrioid histotype.

It shows alterations of specific genes: POLE, PTEN, PIK3R1, PIK3CA, FBXW7, KRAS, and TP53 [13].

The POLE gene encodes the subunit A of DNA polymerase epsilon, which is involved in DNA replication and repair [14]. In EC patients, the most common mutations registered in POLE were P286R and V411L respectively in exon nine and exon thirteen [15].

(2) Microsatellite instability hypermutated

This group is characterized by intermediate prognosis.

It is associated with endometrioid histotype.

It shows alterations of specific genes: PTEN, KRAS, and ARID1A [13].

MSI represents the phenotypic evidence that DNA mismatch repair (MMR) is not functioning normally. MMR deficiency is linked to many cancers such as brain, colon and endometrial. There are four MMR genes (MLH1, MSH2, MSH6, or PMS2) whose inactivation leads to mutations accumulations called MSI. It can occur through several mechanisms: insertions, deletions, point mutations, loss of heterozygosity, copy number changes, structural rearrangements, and methylation of a gene promoter [16].

(3) Copy-number low

This group is characterized by intermediate prognosis.

It is associated to endometrioid histotype.

It shows alterations of specific genes: CTNNB1 and PTEN [13].

Copy number changes are included within the genome structural variation: They comprise duplication or deletion events that have an effect a considerable number of DNA base pairs.

(4) Copy-number high

This group is characterized by unfavorable prognosis.

It is associated with serous histotype.

It shows alterations of specific genes: TP53, FBXW7, and PPP2R1A [13].

High copy variety changes are related to cancer-related genomic instability and fast growth progression and invasion.

### 2.2. PORTEC-4a Classification

The PORTEC-4a is an ongoing trial which is based on molecular risk profiles in EC women. It is focused on the comparison between a molecular-based treatment versus standard adjuvant treatment in early stage EC patients.

It aims to compare the standard treatment according to current international guidelines to the experimental treatment based on genetic risk factors in patients with early stage EC. The actual standard treatment hinges on vaginal BRT, which entails internal radiation of the vaginal vault, using a vaginal cylinder.

Patients are randomized to one of the two arms: molecular profile-based group (experimental one) versus standard recommendations group (the active comparator one). Patients randomized to the molecular profile-based group are followed for vaginal recurrence after surgery, if classified as (i) “favorable molecular risk profile”; they are treated with vaginal BRT, if classified as (ii) “intermediate molecular risk profile”; finally, they are treated with EBRT, if classified as (iii) “unfavorable molecular risk profile”. The primary endpoint of the trial is the vaginal recurrence while the secondary endpoints are occurrence of adverse events, patient quality of life, site of recurrence, progression-free survival, overall survival, and EC-related healthcare costs [10].

The authors reported three prognostic categories as follows:

(1) Favorable:

POLE mutation or No Specific Molecular Profile (NSMP) while not CTNNB1 mutations.

(2) Intermediate:

Mismatch repair-deficient (MMRd) or NSMP with CTNNB1 mutations.

(3) Unfavorable:

LVSI TP53 abnormal immunohistochemical staining or L1CAM overexpression.

CTNNB1 gene encodes beta-catenin protein, which affects cell growth, differentiation and motility. Its mutation is related to carcinogenesis in several tumors depending on the Wnt signaling pathway and is related to EC favorable prognosis [17].

P53 gene encodes for the tumor suppressor p53 protein (TP53) mainly involved in the cell-cycle checkpoints regulation and DNA repair, preserving genomic stability from different type of damages, including senescence and apoptosis; it is altered in many neoplasms [18].

TP53 abnormal immunohistochemical staining is related to EC poor prognosis: Loss of tumor suppressor p53 would lead to a high degree of genomic instability and fast cancer progression and invasion.

L1CAM is a transmembrane protein belonging to the gamma globulin family, which can promote aggressive tumor biology. It has a role in metastasis formation regulating cells migration, invasion, and proliferation [19]. It is associated with more aggressive EC histologies, advanced stage, LVSI, and recurrence [20].

## 3. The Epigenetics of EC

According to online databases such as UniProt, the human genome harbors approximately 20.3 thousand protein-coding genes. However, its length is ca. 3.2 billion base pairs per haploid genome, indicating that the vast majority of the human DNA currently has no known function [21,22]. Thus, in the last decades, ever greater importance has been attributed to the non-protein-coding portion of the genome, and on how it controls several aspects of the cell homeostasis.

As a general rule, with the term “epigenetics” scientists refer to a set of gene function alterations that are mostly mediated either by the action of non-coding RNAs (ncRNA) that may control the half-life and/or the translation of target mRNA, or by structural DNA modifications (such as DNA methylation or histone modifications) mostly impairing DNA transcription; in both cases, although the DNA sequence is not altered, these modifications are inheritable [23]. It is now widely accepted that these phenomena play a pivotal role in cancer etiopathogenesis and, in many instances, they have a high diagnostic and prognostic value [24,25]. This is true also for EC, for which both mechanisms have been amply described in the literature [11,26,27,28,29,30,31].

### 3.1. ncRNA Role in EC Etiology

As for ncRNA, a major classification is made on the basis of their length. Those longer than approximately 200 nucleotides (nt) are called long ncRNA (lncRNA), while the others, that usually span only a few tens of nucleotides (mostly ca. 20–25 nt long), are collectively called short ncRNA (sncRNA). Still, both classes are made up of very heterogeneous molecules in length, function or structure [32]. For example, sncRNA of 20–30 nt include siRNA, miR and piRNA, with different biogenesis and function; on the other hand, YRNA, T-UCR, circRNA, snRNA and snoRNA span lengths of 20–1600 nt, thus partly overlapping sncRNA length with the same degree of heterogeneity in their biology. Consequently, this “structural” classification may not be the most adequate categorization for these molecules. However, due to their widespread use, we will still rely on it in our dissertation, and in this specific case we will refer to lncRNA as to 100+ nt molecules, and to sncRNA as to microRNA (miR).

An analysis of the PubMed database, using the search string “endometrial cancer AND ncRNA”, retrieves almost 750 articles to date. Interestingly, in the time frame from January 2018 to January 2021, the search allows to collect around 250 articles, making up roughly 33% of the total. This means that over the past few years EC research focused on these molecules has grown quite considerably, which reflects their enormous potential in the characterization of this tumor. These studies report that, in EC, these molecules are either up- or down-regulated compared to controls and according to their role as oncogenes or oncosuppressors, either if they promote or inhibit cancer growth and survival, respectively. Overall, it is not always possible to define a given ncRNA (and this is especially true for miR) as either a pure oncogene or as an oncosuppressor, because their targets may vary according to tissue type or stage of development; therefore, it is not infrequent to find that one ncRNA—and especially miR—is up-regulated in one cancer and down-regulated in another one. To further complicate this scenario, even inside the same tumor it is possible to find apparently contradictory results, that in some instances may be explained by the different system used (cell culture vs. biopsy specimen, samples showing different tumor staging, and so on). Consequently, the need to better understand the identity and behavior of each of these molecules is pivotal in understanding EC etiology.

**Figure 1:**
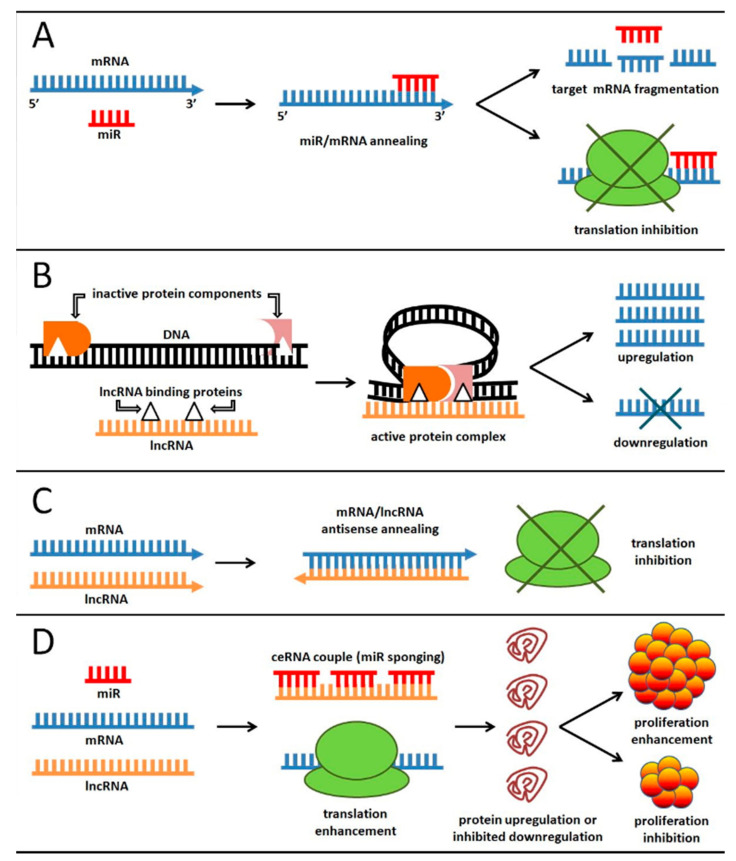
Schematic representation of the most common ncRNA functions in cells. In all panels, the target genes may be either oncogenes or oncosuppressors, depending on the ncRNA involved and the mRNA target; as such, any of the described mechanisms may enhance or inhibit cell proliferation. (**A**): miR-mediated post-transcriptional control of gene expression; miR can pair with target mRNA through sequence homology and promote either mRNA fragmentation or translation impairment. (**B**): gene expression control of lncRNA through DNA structure modification; in this case gene expression is regulated at the transcriptional level. (**C)**: gene expression control of lncRNA through antisense annealing; in this case gene expression is regulated at the post-transcriptional level. (**D)**: lncRNA and mRNA compete for the miR binding; in this case the lncRNA may sponge the miR(s) and allow mRNA translation. See text for additional explanations. Blue, mRNA; red, miR (sncRNA); orange, lncRNA; black, DNA; green, ribosome; other colors/forms, proteins. List of abbreviations: sncRNA is short non coding RNA; miR is micro RNA; lncRNA is long non coding RNA; ceRNA is competing endogenous RNA; mRNA is messenger RNA.

An overview of the mechanisms of action of ncRNA in human cells is summarized in Figure 1. The action of a sncRNA is usually quite straightforward (Figure 1A): It has the capability to bind a target mRNA—usually, its 3′UTR end—through sequence homology and to inhibit its function, either by promoting mRNA degradation or by impairing its translation. As such, sncRNA that inhibit the action of oncogenes are functionally oncosuppressors, and vice versa. Instead, the function of lncRNAs is usually more complex. In some instances, it involves the binding of a sncRNA, thus creating a ceRNA couple (see next section). However, additional genetic control pathways had been described to date, involving the interplay with the DNA double helix either directly (to change its shape and promote regulatory proteins recruitment) (Figure 1B) or indirectly (through interaction with histone modifiers such as HDACs). In those cases, lncRNAs may bind specific proteins causing DNA bending thanks to their interaction with other DNA binding proteins. This promotes their proximity and, in turn, the formation an active complex, able to locally influence DNA transcription. This influence may either enhance or inhibit gene transcription, depending on the DNA regions and proteins involved. Another possible mechanism some lncRNA use to control gene expression is to directly inhibiting target mRNA translation through antisense annealing (Figure 1C).

As a consequence, their target gene(s) (when known) is/are deregulated as well. Several groups focused on the identification of these ncRNAs, either using deep sequencing from EC samples, or by using a bioinformatics approach, allowing the identification of hundreds of putative candidates [12,26,27,31] that are either up- or down-regulated in the considered samples. In such cases, future works will have to figure out whether this deregulation is merely a consequence of an altered tumor cell metabolism, or whether it is causally related to carcinogenesis. According to available databases, there are more than 60 lncRNAs and over 120 miRs deregulated in EC, for which a molecular and/or functional characterization is available, which has shed a light on their possible role as EC biomarkers and possibly, even as therapeutic targets. An overview of the current state of affairs in that regard has been outlined in Table 1 (lncRNA) and Table 2 (miR). Excerpts of these Tables are reported in Table 3, in which we list ncRNAs interacting with genes identified by TCGA or PORTEC4a classifications (see Section 2) and important for EC diagnosis and prognosis.

The above lists are likely to be further expanded in the next few years, which will likely provide a more comprehensive picture of EC etiology. However, available data are already enough to draw some conclusions. Firstly, the target proteins altered in EC (Column 5 in Table 1, and Columns 4 and 5 in Table 2) usually do not carry mutations in their coding sequence. This means that the direct sequencing of the DNA of these genes might bring to inaccurate molecular diagnosis because of the lack of DNA alterations. Such conclusion is equally true for the genes identified by the classification schemes used to date and described in Section 2 (Table 3). Indeed, the deregulation caused by ncRNA occurs mainly at the post-transcriptional level, leaving the DNA untouched. As a consequence, also the molecular diversity of EC in different patients is highly underestimated, potentially bearing to inefficient treatments. This brings to the second conclusion, most ncRNA characterized to date affect only a limited number of cell functions (Column 3 in Table 1 and Table 2) that are nonetheless central in cell replication and survival. This is, at the same time, both a strength and a weakness of these studies. A strength, because having only specific functions altered, it allows to potentially select specific therapeutic targets. For example, in many instances, some proteins are involved more frequently than expected, such as PTEN, mTOR, AKT, MMPs, PI3K, MAPK, Notch, cadherins, and vimentin. These are proteins that are often altered in many cancers, suggesting that efficient ways to impair their function in other tumor types might hint to a good strategy for treating EC as well. The weakness is represented by the fact that, at least in some of them, and especially for miR, there are multiple targets hit at the same time, either directly or indirectly, which amplify the effects of the deregulated ncRNA inside tumor cells. Thus, focusing on only one target protein may not be sufficient to treat EC efficiently, because some metabolic pathways are redundantly altered. Moreover, in this case, a comprehensive analysis of these molecules is the way to pursue towards personalized medicine, in which each patient is characterized by a specific set of molecular alterations, whose targets are well defined, and for whom drawing a therapeutic strategy would yield better results.

### 3.2. ceRNA: At the Crossroad between Small and Long ncRNA Function in EC

Competing endogenous RNAs (ceRNA) are a relatively recent classification of ncRNA based on functional assays. In the last years it has been repeatedly shown that, beyond acting on protein coding genes, ncRNA may also interact between each other (Figure 1D). The interaction occurs thanks to sequence homology, in a way that a given lncRNA may act as a sponge to inhibit the binding of one or more miR to its/their mRNA target. Here, we will refer to two interacting ncRNA as “ceRNA couples”. This kind of interaction, and its deregulation, is present in several human diseases, including cardiovascular anomalies, neurodegenerative disorders, and various types of cancer [221,222].

The outcome of this competition depends on the intracellular amount of each ncRNA involved, recalling that every lncRNA may sponge different sncRNA at the same time. In case the lncRNA depletes the intracellular content of the inhibiting miR (see also Figure 1A), the target mRNA may be regularly translated, producing its protein and affecting cell growth depending on target gene function. This mechanism may be finely tuned, on the basis of the relative amounts of the three RNA (mRNA, lncRNA, and sncRNA) involved. In recent years, several examples of such an interaction have been recorded in EC as well. Table 1 shows the ceRNA couples so far identified, highlighted by the presence of one or more miR in Column 4. It is worth noting that in these cases, because of their peculiar function, lncRNA and sncRNA always have opposite signs in their expression, i.e., if the lncRNA is up-regulated (over-expressed), the corresponding miR is down-regulated because more lncRNA molecules are available to sponge miR and free up the target mRNA. Consequently, if the target mRNA encodes an oncogene, the up-regulation of the lncRNA makes it an oncogene as well (lncRNA and mRNA have the same sign, both increase or decrease at the same time), while the corresponding, “sponged” miR is functionally an oncosuppressor (inverse sign). Similar but opposite behavior for the other way around. It is important to keep this in mind, when planning for possible therapies in EC patients. This makes the molecular characterization of EC patients even trickier, not just because of the expression sign, the physician should consider, during therapy planning, not only the ncRNA that are deregulated, but also those molecules that interact with them either directly (Table 1 and Table 2, Column 4, Table 3) or indirectly (Table 1 and Table 2, Column 5). In fact, it is evident that the oncogenic or oncosuppressive role of the ncRNA is defined by its target(s), and not merely by its status (up- or down-regulated). Without this global vision of the problem, the risk of therapeutic failure is still considerable. In this perspective, it is clear that the old, dualistic classification of EC as types I and II [7] is no longer tenable, and even the one based on coding gene mutations is substantially lacking for the purpose of personalized medicine. Nonetheless, once understood, this complicated scenario may become a powerful weapon in the hands of skilled therapists, because the resulting therapy will consider not only the metabolic pathway of the identified ncRNA, but also possible redundancies or alternative pathways equally hit.

### 3.3. Structural DNA Modifications in EC

As stated before, epigenetic changes may also affect DNA structure. In this case, we are talking about local changes in the three-dimensional shape of DNA, which alters its binding affinity for transcription factors (TF). This change in TF behavior may increase or decrease target gene(s) expression, depending on the type of modification, functionally akin to a gene mutation. This chromatin remodeling is inheritable, although the DNA sequence of the target gene remains the same. From a chemical point of view, there are two main ways to change the DNA affinity for TF: DNA methylation and post-translational histone modifications.

The chemical modifications occurring directly on DNA mainly involve the methylation of Cytosine and, to a lesser extent, of Adenine. In particular, the most common modification is 5-methyl-cytosine (5-mC, cytosine methylated in C-5 carbon). DNA methyltransferases (DNMTs), which use S-adenosyl methionine (SAM) as the methyl donor, are the proteins involved in this process [223]. These modifications are reversible, thanks to the action of specific enzymes called demethylases. Of course, this process is highly regulated and occurs in the presence of specific consensus sequences, and only if specific biochemical signals are present inside the cell. The main targets of DNMTs are the so-called “CpG islands”, (CpG being the abbreviation for 5′-C-p(hosphate)-G-3′, i.e., cytosine and guanine separated by only one phosphate group). CpGs are sequences enriched in Cytosine and Guanine, placed at the 5′-end of genes, involved in the transcriptional control of the downstream gene [224]. Once DNA modification is applied, specific DNMTs act to maintain the DNA methylation in place throughout cell cycles, hence the heritability of these changes. Notably, CpG distribution in human genome is not random [225], and alterations in DNMTs function has been associated with several types of cancer [226], including EC [29]. Global genome methylation in normal vs. neoplastic endometrium is different, and the amount of DNA methylation inside the same CpG island may also vary according to cell type, stage, or time (menstrual cycle) [227], indeed, at least four different subtypes of methylation clusters can be recognized in EC [8]. Remarkably, the data shown here have two important points of contact with what has been described in EC previously [29]. Firstly, many of the already described genes affected by altered methylation (PI3K, Wnt, p16, FGF, NRAS, ERK, PARP, etc.) are also direct or indirect targets of ncRNA action, this means that these genes may be responsible of EC development not only because of gene mutation or DNA methylation, but also as a consequence of altered control pathways driven by these RNA. This is in good agreement with what we stated before about the redundancy of genetic function impaired in EC. In other words, the same gene can be hit in different ways to cause neoplastic transformation: mutation of its coding sequence, transcriptional silencing, or post-transcriptional silencing. Thus, understanding “why” a certain gene function is modified in EC has a deep influence on the therapy selection, for example, using a drug inhibiting mTOR may be inefficient if mTOR is mutated (no protein/drug binding), but efficient if a wild type mTOR is overexpressed. Second, in a few cases (miR-143, miR-145, miR-148b, and miR-152, Table 2) DNMTs are directly influenced in their expression by ncRNA, indicating that these small molecules might potentially deregulate tens of genes by altering the methylation profile of endometrium cells. Then, it would be interesting to analyze if these miR targeting DNMTs also have an indirect effect on genes not listed in their indirect targets, such as PTEN (one of the most frequently altered genes in EC hit 15 times in Table 1 and Table 2) or AKT (21 hits) or PI3K (12 hits). Similarly, it would be interesting to analyze those genes which are known to be deregulated in EC upon DNA methylation variation, but which are not (yet?) present in our target list in Table 1 and Table 2, such as MLH1 and MGMT (hypermethylated) or BORIS and PAX2 (hypomethylated) [29]. This represents an additional layer of complexity in EC diagnosis and characterization, which needs to be taken into account in the perspective of personalized medicine.

Post-translational modification of histones is equally efficient in changing TF affinity for target DNA sequences. Histones are highly conserved proteins that represent the initial and lower organization of chromatin. There are four core histones, namely H2A, H2B, H3, and H4, which assemble in two identical tetramers that make up an octamer around which DNA winds. Chemically, the interaction with DNA, charged negatively due to phosphate groups (acid), occurs because these proteins are positively charged (basic). The number of negative groups on DNA is fixed, but the number of charges on histones may be modified, by adding or deleting additional groups to their amino acids, especially at their N-terminal tail. The modifications include acetylation, methylation, phosphorylation, ubiquitylation, sumoylation, ADP ribosylation, deimination, proline isomerization, crotonylation, propionylation, butyrylation, formylation, hydroxylation, and O-GlcNAcylation [228,229]. The complex changes—which may be present in several combinations and number, allowing scientists to define a “histone code” for chromatin structure—are capable of fine-tuning the gene expression in higher eukaryotes. These modifications change the affinity of histones with DNA, allowing to modify the tightness of the chromatin packing, and in turn the accessibility of TF to the promoter regions of the genes. This, of course, in addition to the “normally” occurring mutations in histone genes, which may be oncogenic as well [230]. The most active and best characterized histone modification proteins are histone acetyltransferases (HATs, which add acetyl groups to amino acids, mainly lysine), histone deacetylases (HDACs, which remove acetylation), histone methyltransferases (HMTs, which add methyl groups to amino acids, mainly lysine and arginine) and histone demethylases (HDMs, which remove methylation). Interestingly, these modifications resemble the behavior of those occurring on DNA, in that they vary in endometrium according to cell type, stage, and time [30]. The role of these proteins in EC has been at least partially expounded upon in several papers (see Reference [230] and references therein). As a general rule, acetylation opens up the chromatin structure, promoting gene transcription, while methylation action depends on the number and position of the modification, thus can either promote or repress gene function. HATs are frequently overexpressed in cancer tissues including EC, while HDACs act in the opposite way. Instead, the oncogenic action of HMTs and HDMs depends on the role they exert in a specific context [214]. The most studied in EC is EZH2, which methylates H3K27 residue. This protein is under the control of both lncRNA (NEAT1, which is a ceRNA on EZH2 mRNA with miR-144-3p and miR-146b-5p, and PCAT1) and sncRNA (miR-101, miR-101-3p, miR-137, and miR-26a). In addition, four other proteins involved in these processes can be found: CHD7, involved in histone acetylation, under the control of the ceRNA couple LINC01410/miR-23; LSD1/KDM1A, a demethylase under the control of miR-137 (together with EZH2); KDM5B/JARID1B, another demethylase, under the control of miR-29c-3p; and HDAC6, a deacetylase, under the control of miR-206. It is apparent that these processes are also under the control of several and redundant ncRNA, indicating that for these processes a set of conclusions similar to those for DNA methylation can be drawn.

## 4. Discussion

The cornerstone of the oncological treatment improvement is to avoid therapeutic side effects while maintaining a safety radicality, to ensure the best possible prognosis for cancer patients.

The actual risk stratification of EC patients is mainly based on clinical–pathologic factors [5], emerging studies are suggesting the inclusion of additional genetic features to better define the prognosis of patients affected by EC, but they are not yet used in the present clinical decision-making process [8,10].

Adjuvant treatments and patients’ surveillance after surgical staging for EC is currently based on stage, clinical variables, and histological variables [5].

Genetic prognostic features are currently the subject of experimental studies that will guide the use of more or less aggressive treatments [10].

Currently, well-defined prognostic elements for a favorable prognosis are considered stage I, endometrioid histologic subtype, no LVSI, G1, POLE mutations, and CTNNB1 mutations [5,8,10].

Prognostic elements for an unfavorable prognosis are considered to be advanced stage, non-endometrioid histologic subtype, LVSI, G3, copy number high, p53 mutations, and L1CAM overexpression [5,8,10].

EC is a very heterogeneous disease characterized by different histotypes and multiple genetic alterations. The molecular diversity of EC in different patients is underestimated, potentially leading to the risk of under or overtreatment. For this reason, the results are sometimes confusing, with some patients classified as low-risk having an unfavorable course of the disease, while some others with high risk factors showing a long progression-free survival.

Our analysis supports this vision: A growing list of hundreds of ncRNAs exists, all of them are deregulated in EC and may be extremely helpful in characterizing EC subtypes. Moreover, for a number of them, also a functional characterization is available, albeit sometimes partial, suggesting that the molecular complexity of EC is still far from being fully clarified. The recent evidence of this complexity, exponentially increasing in the last years, shows that a mere list of deregulated molecules is not sufficient for physicians, they need to rely on a full vision of the cross-interactions between the genome and the epigenome as well, in order to select the most effective therapeutic strategy. As such, the more information can be obtained by patients’ specimen, the better. The development of diagnostic tools capable of analyzing hundreds of ncRNAs at the same time from small biopsy samples is not only technically feasible, but also desirable.

Recently, ncRNAs have been studied with high throughput sequencing aimed at identifying their expression profiles in EC patients and to define their function in EC progression [12].

Several studies have pointed to a connection between ncRNAs and EC prognosis [12,31,63,74,78,98,104,134,163,166,219,231,232,233,234,235], however, such findings derive from dishomogeneous population samples.

Among the authors that show a correlation between ncRNAs and survival rates, Zhou and collaborators reported the prognostic value of a promising predictive model based on the expression of 11 lncRNAs associated with EC patients’ survival data [231].

Ahsen and coworkers have shown a correlation between miRNA and lymph node metastasis [236].

Identifying prognostic biomarkers would be essential to reduce EC recurrence and mortality rates. To that end, we have herein proposed to create a specific panel, to be used with highly parallel genome sequencing, for searching protein coding genes mutations, and possibly regulators of the otherwise wild type genes as well. In this panel we may imagine to put a set of genes with a recognized function in EC, plus the ncRNAs known to control those genes (Table 1, Table 2 and Table 3), plus those markers whose function is not yet known, but that show high diagnostic value. The substantial complexity of the information obtainable from such an analysis will most likely allow for a far deeper diversification of patients’ molecular profile, and aid for a more specific, personalized approach in EC treatment. We summarize these classes of molecules in Table 4.

In addition, we believe that it would be also very interesting to investigate additional potential ncRNA markers that, to date, have not been explored in this cancer, such as YRNA, which might highlight additional actors in EC etiology and would suggest novel potential therapeutic targets [238]. The genetic and epigenetic characterization of patients could lead to surgical strategies (lymphadenectomy versus sentinel lymph node sampling versus total hysterectomy and bilateral salpingo-ophorectomy), adjuvant therapies (BRT versus EBRT versus chemotherapy), and patients’ surveillance (strict follow up versus watchful waiting strategy).

## 5. Conclusions

Overall, ncRNAs seem to show an independent prognostic value, as compared with the well-known clinical variables. With further prospective studies, the ncRNAs could represent valuable biomarkers to improve risk stratification for EC patients.

A prospective and comprehensive analysis of ncRNAs is the way to move towards a personalized medicine, in which each patient is characterized by a specific set of molecular alterations, whose target are well defined, and for whom drawing a therapeutic strategy would likely lead to better results.

## Figures and Tables

**Table 1:** A summary of lncRNAs that play a role in endometrial cancer (EC) pathogenesis and for which a functional characterization is available.

Long non Coding RNA Name	Expression Level	Described Functions	miR Interactions	Other Functional Interactions in EC	References
ABHD11-AS1	up	a, b		cyclin D1, CDK1, CDK2, CDK4, Bcl-xl, VEGFA,p16	[33]
AL161431.1	up	a	miR-1252-5p	MAPK	[34]
ASlnc04080	up	a, b		at least 19 genes	[35]
BANCR	up	b		MMP1/2, ERK, MAPK	[36]
C2orf48	up	n/a	miR-183	CCNB1	[37]
CARLo-5	up	a, c		CDK, MMP2/9,	[38]
CCAT1	up	n/a	miR-181a-5p		[39,40]
CCAT2	up	a, c	miR-216b	Bcl-2	[41]
CDKN2B-AS	up	d	miR-125a-5p	Bcl-2, MRP4	[42]
CHL1-AS1	up	n/a	miR-6076	CHL1	[43]
circ_0002577	up	a, b, e	miR-197	CTNND1	[44,45]
circ_0061140	up/down	a	miR-149-5p	STAT3	[46]
DANCR	up	b	miR-214		[47]
DCST1-AS1	down	c	miR-92a-3p	Notch1	[48]
DLEU1	up	b, e	miR-490	Bcl-2, BAX, E-cadherin, N-cadherin, Snail, vimentin, CASP-3, SP1, PI3K, AKT1, p70S6K, rpS6, GSK3B, STAT3, Bcl-xl, mTOR	[49,50]
FER1L4	down	c		PTEN, AKT	[51,52]
FRMD6-AS2	down	a, c		FRMD6	[53]
GAS5	down	b	miR-103, miR-222-3p	p27, PTEN	[54,55]
H19	up	a, b, c, e	miR-20b-5p, miR-124-3p, miR-612	HIF-1α, AXL, PCNA, Snail, HOXA10, E-cadherin, ITGB3	[56,57,58,59,60,61]
HAND2-AS1	down	c		NMU	[62]
HOTAIR	up/down	b, f	miR-646	PRB, NPM1, Beclin-1, MDR, P-gp, PTEN, PI3K	[63,64,65,66,67,68]
HOTAIRM1	up	a, c, e		HOXA1	[69]
HOXB-AS1	up	a, c	miR-149-3p	Wnt10b, β-catenin, cyclin D1, c-Myc	[70]
LA16c-313D11.11	down	a, c	miR-205-5p	PTEN, PI3K	[71]
LINC00261	down	a, c	miR-27a, miR-96, miR-153, miR-182, miR-183	FOXO1	[72]
LINC00483	up	b	miR-183, miR-192	CCNB1, GRHL1	[37]
LINC00672	down	d		p53, LASP1	[73]
LINC00958	up	c	miR-761	DOLPP1	[74]
LINC01016	up	n/a	miR-302a-3p, miR-3130-3p	NFYA, SATB1	[75]
LINC01170	up	b		AKT	[76]
LINC01220	up	a, b		MAPK11	[77]
LINC01410	up	a, b	miR-23c	CHD7	[78]
LINC-ROR	up	n/a	miR-145		[79]
LINP1	up	a, c		PI3K, AKT	[80]
lnc-NA	down	a, b		NR4A1	[81]
lnc-OC1	up	b	miR-34a	PD-L1	[82]
lncRNA-ATB	up	a, b, e	miR-126	CASP-3, Sox2, TGF-b, PIK3R2	[83]
lncRNA-HEIH	up	a, d		MAPK	[84]
lnc-XLEC1	down	n/a		MBP-1	[85]
LOC134466	down	b	miR-196a-5p	TAC1	[86]
LOXL1-AS1	up	a, b	miR-28-5p	RAP1B	[87]
MALAT1	down	c, e	miR-200c	TGF-B	[88]
MEG3	down	a, f		PI3K, MEG3, Notch1, Hes1	[89,90]
miR143HG	down	b	miR-125a	p53	[91]
MIR22HG	down	a, b	miR-141-3p	DAPK1	[92]
NEAT1	up	a	miR-361, miR-144-3p, miR-146b-5p	MEF2D, ROCK1, WNT7A, VEGFA, PDE4B, EZH2, STAT3, KPNA4, LEF1, MMP9, c-Myc	[93,94,95]
NIFK-AS1	down	a	miR-146a		[96]
NR2F1-AS1	up	a, b, c	miR-363	SOX4, PI3K, AKT	[97]
PCAT1	up	a, b, c, e		E-cadherin, EZH2, Bcl-2, vimentin, N-cadherin, Bad	[98,99]
PCGEM1	up	a, c, b	miR-129-5p	STAT3, Bcl-2, survivin, VEGFA, MMP2	[100]
PVT1	up/down	a, b	miR-195-5p	UPF1, FGFR1, FGF2	[101,102]
RNA-14327.1	up	a, e		Kca3.1	[103]
RP11-357H14.17	up	n/a	miR-24-1-5p, miR-27b, miR-143, miR-204, miR-503, miR-4770	up to 183 targets	[104]
RP11-395G23.3	down	a, c	miR-205-5p	PTEN, AKT	[105]
RP11-89K21.1	up	n/a	miR-27b, miR-4770, miR-143, miR-204, miR-125a-5p, miR-125b-5p, miR-139-5p, miR-670-3p	up to 183 targets	[104]
SNHG16	up	a	miR-490-3p	HK2	[106]
SNHG5	down	a, c	miR-25-3p	BTG2	[107]
SNHG8	up	a	miR-152	c-MET	[108]
SRA	up	a, b, e		EIF4E-BP1, Wnt, β-catenin	[109]
TDRG1	up	a, b, c		VEGFA, AKT, PI3K, mTOR	[110,111]
TUG1	up	n/a	miR-34a-5p, miR-299		[112]
TUSC7	down	a, e	miR-23b, miR-616	SOCS4	[113,114]
ZNRD1-AS1	up	n/a		ZNRD1	[115]

Note: lncRNAs are listed in alphabetical order (Column 1) and for each we report its expression in EC compared to control (Column 2) (either up- or down-regulated), its role in the development of EC (Column 3) and its functional interactions with target genes in EC (Columns 4 and 5), as reported in the available literature (Column 6). In the case of no data being available, we report “n/a”. In Column 3, the following abbreviations are used: a, cell growth; b, apoptosis; c, cell invasion/migration; d, drug resistance; e, EMT transition; f, other. The interactions with miR are highlighted in Column 4, because in those cases the lnc/snc couple acts as ceRNA (see text for further explanations), while in Column 5 we report the protein coding genes. As for the latter targets, for simplicity, we pooled together both up- and down-regulated genes, as both are “deregulated” in EC compared to controls; we included all proteins cited in the respective references, even if their regulation is not a direct effect of the ncRNA. Data in the table are mostly retrieved from http://www.bio-bigdata.com/lnc2cancer/ (accessed on 13 January 2021) and updated according to the most recent (1/2018-onward) data available in PubMed (http://pubmed.ncbi.nlm.nih.gov/; last accession: 28 January 2021). Notes: (i) data about lncRNA CTBP1-AS2 are not included, because the article was retracted, due to ethical issues; (ii) data about lncRNA OGFRP1 are not included, because the article was retracted, due to methodological issues; (iii) data about lncRNA HOTTIP are not included because the article was retracted, with no explanation available.

**Table 2:** A summary of sncRNAs that play a role in EC pathogenesis and for which a functional characterization is available.

miR Name	Expression Level	Described Functions	Primary Targets	Secondary Targets	References
miR-101	down	a, b, c, f	EZH2, MCL-1, FOS, mTOR, COX-2	VEGF-A, TSP-1, COX-2, PGE2, P450arom	[116,117,118]
miR-101-3p	down	f	EZH2		[119]
miR-103	up	a	ZO-1		[120]
miR-106a	up	a, b, c	MYC, BCL2L11	p21, BIM	[121,122]
miR-106b	up/down	a, b	PTEN	AKT, mTOR	[123,124]
miR-107-5p	up	a, c	ERα		[125]
miR-10b	up	a, b, c	HOXB3		[126]
miR-1271	down	a, c, b	CDK1, LDHA		[127,128]
miR-130b	down	e	ZEB1		[129]
miR-134	down	a, c	POGLUT1, Notch		[130]
miR-137	down	a	EZH2, LSD1		[131]
miR-139-5p	down	a, c	HOXA10		[132]
miR-142	down	a	CCND1	Ki67	[133]
miR-143	down	a, c, f	DNMT3B, MAPK1		[134,135]
miR-145	down	f	DNMT3B, OCT4		[134,136]
miR-145-5p	down	a, c, b	DUSP6		[137]
miR-148b	down	a, c, e, f	ERMP1, DNMT1	HIF-1, Nrf2	[138,139]
miR-152	down	a, b, f	DNMT1, E2F3, MET, Rictor, SOS2, NRAS, APC, PIK3R3, SOS1, PTEN, CDC25B		[140,141,142]
miR-155	up	a	AGTR1		[143]
miR-15a-5p	down	a	WNT3A		[144]
miR-181c	down	b	PTEN, NOTCH2	Bax, Bcl-2, AKT, p53, Cyclin D.	[145,146]
miR-181d	up	a, b, f	PIK3R3, SOS1, PTEN		[141]
miR-182	up	a	TCEAL7	c-Myc, cyclin D1, NFκB	[147]
miR-183	up	a, b, c, e	MMP9, CPEB1	E-cadherin, vimentin	[148,149]
miR-183-5p	down	a, b, c, e	Ezrin		[150]
miR-184	down	c	CDC25A	NOTCH1/2/3/4, HES1	[151]
miR-191	up	a	TET1		[152]
miR-195	down	c, e	SOX4, GPER	TIMP-2, MMP2/9, PI3K, AKT	[153,154]
miR-200a	up	e	FOXA2	E-cadherin, vimentin	[155]
miR-200b	up	c	TIMP2	MMP2	[156]
miR-200c	up	a, c, e	BRD7, BMI-1, PTEN, PTENP1	β-catenin, cyclinD1, c-myc, AKT, Slug, N-cadherin, PI3K, E-cadherin	[157,158,159]
miR-202	down	c, e	FGF2	β-catenin, N-cadherin, vimentin, E-cadherin	[160]
miR-204	down	a, c	FOXC1		[161]
miR204-5p	down	a, c	TrkB, SF3B1, FBXW7, BRD4		[162,163]
miR-205	up	a, b, c, e	ESRRG, PTEN, AKT	E-cadherin, Snail	[164,165,166,167,168]
miR-206	down	a, c	HDAC6	PTEN, AKT	[169]
miR-21-5p	up	e	SOX17		[170]
miR-215	up	a, c, d, e	LEFTY2		[171]
miR-218	down	c	ADD2		[172]
miR-222-3p	up	a, c, d	ERα		[173]
miR-223	down	a	IGF-1R		[174]
miR-25	up	a, b	p21, BIM		[121]
miR-26a	down	e	EZH2	N-cadherin, Vimentin, Snail, E-cadherin	[175]
miR-27a-5p	up	c	SMAD4		[176]
miR-27b-3p	down	c	MARCH7	Snail, Vimentin, E-cadherin	[177]
miR-29a-5p	down	a, c, b	TPX2		[178]
miR-29b	down	a, c, d	PTEN	BAX, Bcl-2, AKT	[179]
miR-29b	down	f	VEGFA	MAPK, PI3K, mTOR, Bcl-2	[180]
miR-29c-3p	down	d	KDM5B		[181]
miR-301b	down	e	ZEB1		[129]
miR-302a-5p	down	c	HMGA2		[182]
miR-30c	down	a, c	MTA1	mTOR, 4E-BP1, AKT	[183,184,185]
miR-320a	down	a, c, e	eIF4E, IGF-1R	MMP3, MMP9, TGF-β1	[186,187]
miR-326	down	a, c, e, f	GPR91, TWIST1	STAT3, VEGF	[188,189]
miR-335	up	a	RBM10	Numb-L	[190]
miR-340	down	a, b	p27, KIP1, Bax, Casp3		[191]
miR-340-5p	down	c, e	eIF4E	MMP3, MMP9, TGF-β1	[186]
miR-34a	down	a, c, e	L1CAM, p16, Ki-67, Notch1		[192,193,194]
miR-34c	down	a, b, c, d	E2F3		[195,196]
miR-367-3p	down	c	HMGA2		[182]
miR-372	down	a, c	RhoC, Cyclin A1, CDK2	MMP2, MMP9, PARP, Bax	[197]
miR-373	up	a, c, e	LATS2	Wnt	[198]
miR-381	down	a, c	IGF-1R	AKT, ERK	[199]
miR-409	down	a	Smad2		[200]
miR-424	down	a, b, e	E2F7, GPER, IGF-1R, CPEB2	PI3K, AKT, E-cadherin, vimentin	[201,202,203,204]
miR-449a	down	a, b, c	CDC25A, NDRG1, SRC	PTEN, AKT, ERK1/2	[205,206,207]
miR-494-3p	up	a, c	PTEN	PI3K, AKT	[208]
miR-495	down	a, b, f	GSK3B, NRAS, TCF4, PIK3CB, PIK3R3, CCND1, AXIN2, PIK3R1, SOS1, PIK3CA, FOXO3, PTEN	Bcl-2, VEGF, Bax, CASP-3	[141,209]
miR-505	down	a	TGF-α	MMP2, MMP9, CDK2, Bax, PARP	[210]
miR-522	up	a, c	MAOB		[211]
miR-543	down	a, c	FAK, TWIST1		[212]
miR-589-5p	down	a, c	TRIP6	E-cadherin, N-cadherin, vimentin	[213]
miR-652	up	a, c	RORA	β-catenin	[214]
miR-93	up	a, b, e, f	p21, BIM, FOXA1	E-cadherin, N-cadherin, MAPK1, RBBP7, Smad7	[121,215,216]
miR-93-5p	up	a, c	IFNR1	STAT3, MMP9	[217]
miR-940	up	a, c	MRVI1		[218]
miR-944	up	a	CADM2		[219]
miR-99a	down	a, b, c	AKT1, mTOR		[220]

Note: sncRNA (miR) already cited in Table 1 are omitted from this list. miR are listed according to increasing identification number (Column 1) and for each we report its expression in EC compared to control (Column 2) (either up- or down-regulated), its role in the development of EC (Column 3) and its functional interactions with primary and secondary target genes in EC (Columns 4 and 5) as reported in the available literature (Column 6). In the case of no data being available, we report “n/a”. In Column 3, the following abbreviations are used: a, cell growth; b, apoptosis; c, cell invasion/migration; d, drug resistance; e, EMT transition; f, other. In Column 4, primary targets are the genes whose function is directly controlled by the miR in EC, while in Column 5 secondary targets are those genes whose function in EC is influenced by the action of primary targets. For simplicity, we pooled together all targets independently of their up- or down-regulation, as all are “deregulated” in EC compared to controls; we included all deregulated proteins cited in their respective reference(s) (Column 6). Data in the table are primarily retrieved from http://mircancer.ecu.edu/ (accessed on 15 January 2021) and updated according to the most recent (1/2018-onward) data available in PubMed (http://pubmed.ncbi.nlm.nih.gov/; last access: 28 January 2021).

**Table 3:** ncRNA which functionally interact with EC diagnostic and prognostic genes.

Gene (TGCA/PORTEC4a Classifications)	Interacting lncRNA	Interacting sncRNA
CTNNB1 (β catenin)	HOXB-AS1, SRA	miR-200c, miR-202, miR-652
FBXW7	n/a	miR-204-5p
L1CAM	n/a	miR-34a
PIK3CA	n/a	miR-495
PIK3R1	n/a	miR-495
PTEN	FER1L4, GAS5, HOTAIR, LA16c 313D11.11, RP11-395G23.3	mir-106b, miR-152, miR-181c, miR-181d, miR-200c, miR-205, miR-29b, miR-494-3p, miR-495
TP53	LINC00672, miR143HG	n/a
ARID1A, KRAS, MLH1, MLH2, MLH6, PMS2, POLE, PPP2R1A	unknown	unknown

Note: Reported genes are those indicated as diagnostic/prognostic markers in either TCGA or PORTEC4a classification, they are listed in alphabetical order (Column 1). ncRNAs are divided in long (Column 2) and short (Column 3) ncRNA. In Column 3, only miR which directly interact with the target gene (Column 1) are reported, for indirect interactions, see Table 2. Here, ncRNAs in the same Table row do not necessarily interact with each other, forming ceRNA couples, for this information and for the bibliographic references supporting these data, we redirect the reader to Table 1. For simplicity, genes for which no known interacting ncRNA is identified to date are collectively listed in the last row of the table; n/a, data not available.

**Table 4:** Proposal for a diagnostic panel for highly parallel sequencing approach.

Target	Purpose	Expected Analysis Output	Candidate Genes	References
Coding gene	Finding gene mutations	Sequence mutation(s)	PTEN, VEGF, TP53, FGF, PIK3CA, Ki-67, β-Catenin, EGFR, RAS-RAF-MEK-ERK pathway, p21, p16, ERBB2, E-Cadherin, ER, PR, Cox-2	[237]
lncRNA (a)	EC marker and identification of potential target genes	Up-/down-regulation	See Table 1 (62 lncRNA)	See Table 1
sncRNA (a)	EC marker and identification of potential target genes	Up-/down-regulation	See Table 1 and Table 2 (127 miR)	See Table 2
lncRNA (b)	EC marker	Up-/down-regulation	*ENSG00000260684, ENSG00000229589, ENSG00000224037, ENSG00000235499, ENSG00000224905, ENSG00000260992, ENSG00000248008, ENSG00000234945, ENSG00000182648, ENSG00000253636, ENSG00000233760*	[231]
sncRNA (b)	EC marker	Up-/down-regulation	Several tens	[12,26,27,31,236,237]

Note: Modern technologies allow us to put all of these molecules inside one or two chips and to have a complete analysis of EC samples in a few hours, with the advantage of a much better molecular characterization of the patient. Notes: (a) ncRNA with known function (see Table 1 and Table 2), (b) ncRNA with unknown function but high diagnostic value.

## Data Availability

Not applicable.
